# Rapid Development of Propofol Infusion Syndrome After Short-Term Exposure

**DOI:** 10.7759/cureus.105134

**Published:** 2026-03-12

**Authors:** Nancy M Boulos, Athreya Steiger

**Affiliations:** 1 Anesthesiology and Perioperative Medicine, University of California, Los Angeles, Los Angeles, USA

**Keywords:** critical care, postoperative outcomes, propofol infusion syndrome, propofol toxicity, sedation

## Abstract

We describe a 34-year-old man with well-controlled epilepsy and severe autism spectrum disorder (ASD) complicated by self-injurious behaviors who developed green urine, hypertriglyceridemia, and metabolic acidosis concerning for propofol-related infusion syndrome (PRIS) in less than 48 hours of sedation with propofol following left-eye scleral buckle surgery. The patient required multimodal and continuous sedation to prevent postoperative trauma to the surgical site. The patient required a dexmedetomidine infusion at 1.5 mcg/kg/hour and a fentanyl infusion maintained between 50 and 100 mcg/hr in addition to propofol maintained at 70-100 mcg/kg/min. Approximately 24-30 hours after the initiation of a propofol infusion at 80-100 mcg/kg/min, he developed green urine without evidence of infection. Laboratory evaluation showed a rise in serum triglycerides from 305 mg/dL to 569 mg/dL overnight. Despite no initial acidosis, reduction of the propofol dose was followed by the onset of profound metabolic acidosis (values: pH 7.36, HCO₃ 15.8, pCO2 29) and creatine kinase (CK) elevation to 4,283 U/L. The constellation of findings raised clinical concern for early or atypical PRIS. This case highlights the diagnostic challenges of distinguishing early PRIS manifestations in patients requiring unusually high and sustained sedation and underscores the importance of vigilance even at propofol doses typically considered safe.

## Introduction

Propofol is widely used for intensive care unit (ICU) sedation but carries a rare risk of propofol-related infusion syndrome (PRIS), classically characterized by metabolic acidosis, rhabdomyolysis, hyperlipidemia, cardiac dysfunction, and renal failure [1]. Although PRIS typically appears at doses >5 mg/kg/hr (≈83.33 mcg/kg/min) for >48 hours, cases at lower exposure levels and shorter durations have been reported [1].

Early manifestations may be subtle and nonspecific, which can delay recognition before the development of life-threatening complications. We present a case of suspected early PRIS in a patient with severe autism spectrum disorder (ASD), extreme self-injurious behavior, and complex psychopharmacology requiring prolonged ICU sedation. This case highlights the diagnostic challenges in such patients and underscores the importance of recognizing early laboratory abnormalities, including rising triglycerides and creatine kinase (CK), in conjunction with clinical findings during propofol sedation.

## Case presentation

Patient information

A 34-year-old, 73-kg man with American Society of Anesthesiologists (ASA) physical status III, severe ASD with marked self-injurious behavior (up to 800 episodes/day), and epilepsy well controlled on levetiracetam was admitted after undergoing left scleral buckle and cryotherapy for repair of a self-inflicted ocular injury.

Home medications included escitalopram, guanfacine, mirtazapine, propranolol, risperidone, lorazepam, and levetiracetam (Keppra).

Perioperative course

As the patient was known to have extreme agitation with self-injurious behavior, both the surgical team and the patient’s medical decision-maker voiced concern that the patient would harm his eye during the critical postoperative period for healing of surgical incisions. After an interdisciplinary discussion with the patient’s medical decision-maker, the psychiatry team, ophthalmology team, and ICU team, the plan was to maintain the patient on a dexmedetomidine infusion in the ICU postoperatively. Upon arrival to the ICU, the patient became extremely agitated despite a maximum-dose dexmedetomidine infusion of 1.5 mcg/kg/hr. He received a total of 4 mg of intravenous (IV) midazolam, 10 mg of IV haloperidol, 50 mg of IV ketamine, and 20 mg of IV propofol, ultimately requiring endotracheal intubation with an additional 180 mg of propofol to facilitate airway management. Per discussion with psychiatry, all the patient’s home medications above were continued as scheduled via nasogastric tube, except guanfacine.

Intraoperative medications

The patient received premedication with 25 mg oral (PO) midazolam and 200 mg intramuscular (IM) ketamine. General anesthesia was induced, and the airway was secured via endotracheal intubation. Anesthesia was maintained with sevoflurane, with a total of 50 mg propofol administered intraoperatively. The total anesthetic duration was approximately 90 minutes. The patient was extubated uneventfully in the operating room while maintained on a dexmedetomidine infusion. 

Clinical course

Twenty-Four to Thirty Hours After Re-intubation

About 26 hours after re-intubation, the patient was noted to have developed green-tinged urine. Urinalysis did not have any evidence of infection. A venous blood gas (VBG) was obtained, which showed pH 7.40, pCO2 42, and HCO₃ 25.8, and triglycerides were 305 mg/dL (increased from 95 mg/dL at the initiation of the propofol infusion). There was no acidosis, lactate elevation, changes in creatinine, or hemodynamics. Laboratory values and reference ranges are highlighted in Table 1. 

**Table 1:** Serial laboratory results following re-intubation Serial venous blood gas measurements and associated laboratory values obtained following re-intubation. All abbreviations are spelled out and units of measurement are provided. Reference ranges reflect institutional standards.

Time after re-intubation	Laboratory test	Result	Reference range
~26 hours	Venous blood pH	7.40	7.30–7.40
	Venous partial pressure of carbon dioxide (millimeters of mercury)	42	37-65
	Venous bicarbonate (millimoles per liter)	25.8	23-31
	Triglycerides (milligrams per deciliter)	305	<150
~42 hours	Venous blood pH	7.36	7.30–7.40
	Venous partial pressure of carbon dioxide (millimeters of mercury)	46	37-65
	Venous bicarbonate (millimoles per liter)	26.5	23-31
	Triglycerides (milligrams per deciliter)	569	<150
~44 hours	Venous blood pH	7.36	7.30-7.40
	Venous partial pressure of carbon dioxide (millimeters of mercury)	29	37-65
	Venous bicarbonate (millimoles per liter)	15.8	23-31
	Lactate (milligrams per deciliter)	12	5-18
	Creatine kinase (units per liter)	4,283	63-473
~48 hours (four hours after the discontinuation of propofol)	Venous blood pH	7.34	7.31–7.41
	Venous partial pressure of carbon dioxide (millimeters of mercury)	47	41–51
	Venous bicarbonate (millimoles per liter)	25.2	22–29
	Triglycerides (milligrams per deciliter)	303	<150

About 16 hours after the green urine was first noted, the patient’s urine was noted to be increasingly dark green. Triglycerides rose to 569 mg/dL. VBG showed pH 7.36, pCO2 46, and HCO₃ 26.5, without a lactate elevation. Due to the concern for the possible development of PRIS, the patient’s propofol was weaned, and a titratable ketamine gtt was started with the goal of cross-titrating off propofol. Given the concerning trends, but continued need for heavy sedation, a midazolam infusion was started, and propofol was further reduced to 50 mcg/kg/min. About two hours later, a repeat VBG showed a profound metabolic acidosis: pH 7.36, pCO2 29, HCO₃ 15.8, lactate 12 mg/dL, and CK of 4283 U/L. His renal function remained stable. Propofol was subsequently rapidly and aggressively discontinued. 

Within hours of discontinuing propofol, the patient’s urine returned to a yellow color. Repeat triglycerides measured four hours after propofol cessation decreased to 303 mg/dL, and a venous blood gas showed resolution of metabolic acidosis (pH 7.34, pCO₂ 47 mmHg, and HCO₃ 25.2 mEq/L). Throughout his ICU stay, his heart rate remained 50-70 beats per minute, and mean arterial pressures (MAPs) were consistently >60 mmHg without vasoactive support. After stopping propofol, his heart rate increased to 90-100 beats per minute, while MAPs remained stable. A notable limitation is the scarcity of documented vitals outside this encounter; however, during anesthesia, heart rates were 50-70 beats per minute, whereas in the few other documented encounters, they were closer to 90-100 beats per minute.

## Discussion

This case highlights the challenges associated with the early diagnosis of PRIS and underscores the importance of prompt recognition of its sequelae to prevent potentially catastrophic outcomes [1,2]. Classic signs and symptoms of PRIS include green urine, rapidly rising triglycerides, new-onset metabolic acidosis, and elevated CK [1,3]. Although the pathophysiology is not fully understood, PRIS is thought to result from the disruption of mitochondrial function through the inhibition of fatty acid oxidation and impairment of the electron transport chain [4]. Propofol has been shown to directly affect Complexes I, II, and III of the electron transport chain, with coenzyme Q serving as the primary site of interaction [5].

In this patient, green urine was the first sign of developing PRIS. This phenomenon is a well-documented but generally benign effect of propofol, attributed to renal excretion of phenolic propofol metabolites [6,1,3]. While green urine alone rarely indicates toxicity, its appearance alongside rapidly rising triglycerides, new metabolic acidosis, and CK elevation served as an early warning signal. Within the context of developing PRIS, green urine may reflect an excessive metabolic burden from propofol [1,7].

Propofol’s lipid-based formulation also commonly leads to hypertriglyceridemia [1,3,8]. In this patient, triglycerides rose from 305 to 569 mg/dL over 24 hours, indicating a significant lipid load and impaired clearance in conjunction with other metabolic abnormalities. Prolonged propofol infusions have been associated with a metabolic shift from oxidative phosphorylation to glycolysis, increased reactive oxygen species production, and activation of apoptotic pathways [4]. They also inhibit palmitoyl carnitine-driven fatty acid oxidation, promoting a physiologic energy crisis that favors anaerobic metabolism and contributes to profound metabolic acidosis. Among patients with PRIS, the incidence of metabolic acidosis has been reported as high as 79.8%, with rhabdomyolysis observed in 26.3% [1,2,8].

Although this patient did not initially exhibit all hallmark features of PRIS, the syndrome can present atypically, particularly early in its course [3,7]. Beyond prolonged high-dose propofol exposure, a history of epilepsy may predispose patients to PRIS. While the exact mechanism remains unclear, some evidence suggests an increased risk in patients with epilepsy, and valproic acid in particular has been implicated as an independent risk factor [9,10]. In this case, however, none of the patient’s home medications, including antiepileptic drugs, are known to increase PRIS risk. Furthermore, while some institutions monitor triglycerides every 48-72 hours in patients on prolonged propofol sedation, this patient’s triglycerides rose within approximately 16 hours of the initial measurement, emphasizing the need to recognize early warning signs.

Alternative diagnoses were considered. Urinary tract infection was unlikely, as complete blood counts and urinalysis showed no evidence of acute infection. Acute rhabdomyolysis secondary to agitation was also improbable given heavy sedation. Serial electrocardiograms did not reveal QTc prolongation, Brugada-like changes, or ischemia characteristic of PRIS. There were no features suggestive of neuroleptic malignant syndrome (e.g., fever, rigidity), and despite SSRI use, serotonin syndrome was unlikely due to the absence of hyperthermia, clonus, or hyperreflexia.

Given the temporal association of metabolic derangements with prolonged propofol infusion and the constellation of clinical findings, PRIS remained the leading diagnosis. Management included initiation of phenobarbital per psychiatry recommendations, continuation of home psychiatric medications, and a dexmedetomidine infusion to facilitate safe extubation in the ICU. Environmental modifications, such as minimizing staff presence and removing unnecessary monitors, were implemented to reduce post-extubation agitation.

## Conclusions

This case demonstrates early and atypical manifestations of propofol-related metabolic toxicity in a patient requiring unusually high sedation for behavioral control. Although propofol is a safe and effective drug for sedation in the critical care setting, it carries the risk of significant metabolic side effects that can have profound consequences if not appreciated promptly. Given the potentially dire consequences of PRIS, clinicians should consider evaluation for metabolic abnormalities associated with PRIS if a patient demonstrates signs of excess propofol metabolic burden, such as green urine. Recognition of green urine, escalating triglycerides, CK elevation, and new metabolic acidosis, especially in the absence of infection or other clear etiologies, should prompt rapid reassessment of propofol exposure and consideration of PRIS. Importantly, as noted above, green urine alone is a benign phenomenon known to occur with propofol metabolism. Clinicians caring for patients with severe neurobehavioral disorders may face unique sedation challenges, and heightened vigilance for propofol toxicity is warranted.
